# Immunological pathogenesis and treatment of systemic lupus erythematosus

**DOI:** 10.1007/s12519-019-00229-3

**Published:** 2019-02-22

**Authors:** Lu Pan, Mei-Ping Lu, Jing-Hua Wang, Meng Xu, Si-Rui Yang

**Affiliations:** 1 Department of Pediatric Rheumatology and Allergy, The First Hospital, Jilin University, Changchun, China; 2 Department of Allergy Immunology and Rheumatology, Children’s Hospital, Zhejiang University School of Medicine, Hangzhou, China

**Keywords:** Immunological pathogenesis, Systemic lupus erythematosis, Treatment

## Abstract

**Background:**

Systemic lupus erythematosis (SLE) is a complex and clinically heterogeneous autoimmune disease. A variety of immunological defects contribute to SLE, including dysregulated innate and adaptive immune response. A clearer understanding of the mechanisms driving disease pathogenesis combined with recent advances in medical science is predicted to enable accelerated progress towards improved SLE-personalized approaches to treatment. The aim of this review was to clarify the immunological pathogenesis and treatment of SLE.

**Data sources:**

Literature reviews and original research articles were collected from database, including PubMed and Wanfang. Relevant articles about SLE were included.

**Results:**

Breakdown of self-tolerance is the main pathogenesis of SLE. The innate and adaptive immune networks are interlinked with each other through cytokines, complements, immune complexes and kinases of the intracellular machinery. Treatments targeted at possible targets of immunity have been assessed in clinical trials. Most of them did not show better safety and efficacy than traditional treatments. However, novel targeting treatments are still being explored.

**Conclusions:**

Dysregulated immune response plays a critical role in SLE, including innate immunity and adaptive immunity. Biologic agents that aim to specifically target abnormal immune processes were assessing and may bring new hope to SLE patients.

## Introduction

Systemic lupus erythematosis (SLE) is a chronic autoimmune disease characterized by the production of autoantibodies and the deposition of immune complexes, affecting a wide range of organs. Genetic factors, environmental factors and hormonal factors are believed to contribute to the occurrence of SLE. However, the pathogenesis of SLE is complex and remains unknown. Breakdown of self-tolerance plays a critical role in the occurrence and development of SLE. Innate and adaptive immune responses against self-antigen induce the production of autoantibodies and the deposition of immune complexes in tissues leads to the activation of complement, accumulation of neutrophils and monocytes, and self-reactive lymphocytes [1]. Despite the improvement in the survival of SLE in the past decades, further improvement in the disease prognosis is hampered by organ damage caused by disease itself and adverse events related to conventional therapies. The in-depth study of disease pathogenesis is helpful to find new therapeutic targets and new biomarkers of SLE. In this review, we summarized recent advances in immunological pathogenesis and related targeting treatment of SLE.

## Dysregulation of innate immunity

### Dendritic cells (DCs)

#### Type I interferon (IFN) in SLE

Recent studies have found a close relationship between type I IFN and SLE [2, 3], especially IFN-α. In more than half of SLE patients, gene expression of the “IFN-α signature” was found in peripheral blood mononuclear cells [4, 5]. IFN-α can activate lymphocyte, DCs and natural killer (NK) cells, thus breaking the autoimmune tolerance. In the case of infection, the dsRNA/ssRNA or dsDNA of virus/bacteria served as exogenous type I IFN attractant to activate the nuclear factor-κB (NF-κB) pathway and then produce IFN-α, which stimulates the immune system to produce autoantibody forms B cells to the nuclear antigen components of apoptotic cells [6]. Proper clearance of apoptotic cells is thought to prevent exposure of self-antigens and inhibit the activation of immune cells. In SLE, however, the rate of apoptosis increased or clearance is suboptimal, leading to an increase in autoantigen–antibody complexes, which have been proved to be endogenous type I IFN inducing agents, and can continue to produce type I IFN, forming a vicious cycle. In addition, IFN-α can promote the activation of T helper (Th) cells, improve the ability of antigen presentation of DCs, and subsequently induce the production of such cytokines as interleukin (IL)-1, IL-2, IL-4, IL-6 and IL-8. It also can affect the expression of mitochondrial genes, change the activity of adenosine triphosphate sensitive K channels, and cause various energy metabolism disorders.

#### Interaction between pattern recognition receptors (PRRs) and DCs

DCs, as antigen presenting cells, can activate primitive T cells and stimulate the proliferation and differentiation of B cells, playing a role in the innate immune system and in the activation of the adaptive response. In SLE patients, IFN-α can promote the transformation of monocytes into DCs, while DCs recognize antigens and continuously produce IFN-α, which in turn circulates and drives the autoimmune response of SLE.

**Fig. 1:**
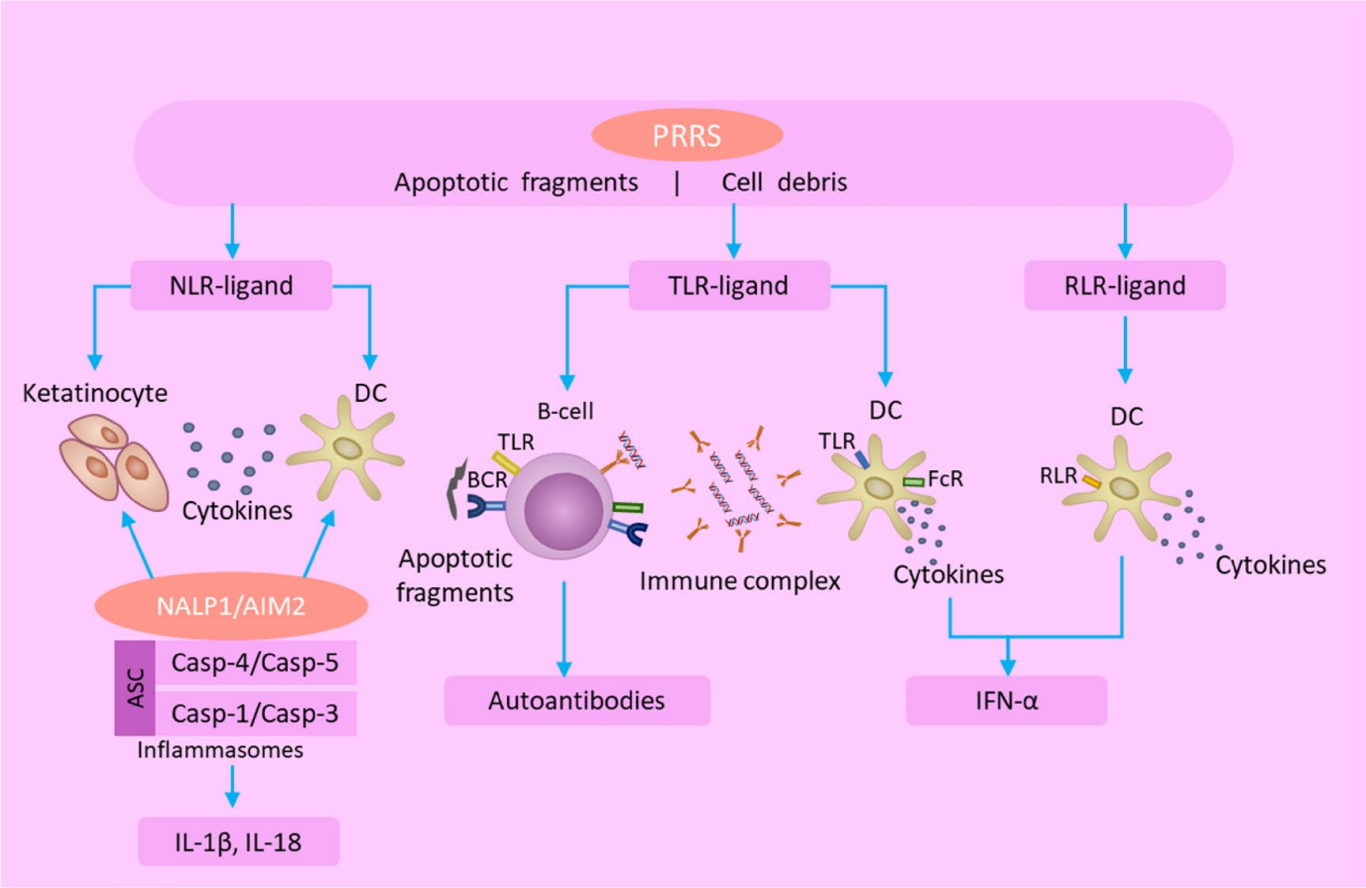
Pattern recognition receptors play a vital role in the innate immunity in lupus. *PRRs* pattern recognition receptors, *NLR* nucleotide binding and oligomerization domain receptors, *TLR* toll-like receptor, *RLR* retinoid acid inducible gene-I-like receptors, *DC* dendritic cell, *BCR* B-cell receptor, *FcR* fragment crystallizable receptor, *NALP1* NAcht leucine-rich-repeat protein 1, *AIM2* absent in melanoma 2, *ASC* apoptotic speck-like protein containing a caspase recruitment domain, *IFN* interferon, *IL* interleukin

The presence of dying cells in lymph nodes and various tissues is a common feature of SLE [7, 8]. Apoptotic cells are removed suboptimally and exposed for prolonged periods of time to form autoantigens, contributing to break the immune tolerance. In the innate immune system, PRRs recognize the apoptotic cell debris or damaged cells and then activate the immune cells. At present, there are at least three distinct types of PRRs: the toll-like receptors (TLRs), the nucleotide binding and oligomerization domain receptors (NLRs), and the retinoid acid inducible gene-I-like receptors (RLRs). TLR plays a key role in the innate immune system. In mouse models, production of antinuclear antibodies depends on endosomal TLRs, which bind dsDNA or ssRNA [9–11]. In SLE patients, TLR9 ligand (such as CpG) can activate plasma cell-like DCs (pDCs) through the interferon regulatory factor (IRF) signaling pathway, producing a large amount of type I IFN (Fig. 1). In addition, the activation of pDCs by TLR7 ligand resulted in increased expression of IL-1β and IL-23, and promoted the differentiation of Th17 (Fig. 1). Furthermore, TLR ligand can also activate B cells and produce autoantibodies to form immune complexes containing ribonucleic proteins and nucleosomes. DCs can identify immune complexes containing chromatin through the surface receptor, FcγIIa, and promote nucleic acid endocytosis, thereby maintain and amplify the immune response. After NLR ligand activates keratinocytes and DCs, different forms of inflammatory ligands are formed and combined with corresponding inflammasomes, such as NAcht leucine-rich-repeat protein/absent in melanoma 2. Then, caspase-1 and caspase-5 are activated, respectively, to promote the production of IL-1β and IL-18. Moreover, incompletely digested DNA existed in mice and humans aggravates the disease by increasing IFN-stimulatory DNA responses (Fig. 1).

## Neutrophils

Neutrophils, critical components of innate immune system, are involved in inflammatory and infectious processes. Improper activation of neutrophils will release protease, tissue damage factors and reactive oxygen species, leading to tissue damage in SLE. Meanwhile, activated neutrophils can release a large number of cytokines and chemokines, leading to immune regulation disorders [12].

In recent years, neutrophils have been found to be involved in autoimmune disease with a new structure: neutrophils extracellular traps (NETs). NETs are fibrous networks assembled from nuclear and granula components which protrude from the membrane of neutrophils that are activated. NETs also contain calprotectin, matrix metalloproteinase 9, lysosomal membrane protein-2. NETosis is the forming progress of NETs. Studies showed that autoantibodies in vasculitis and SLE are components of NETs. Furthermore, NETs are also involved in the sepsis-associated organ damage [13, 14]. The suboptimal clearance of NETs and/or excessive NETs formation is involved in the pathogenesis of SLE [15]. Previous studies revealed that NETs formation and imbalance of NET degradation externalize autoantigens, which induce type I IFN synthesis and endothelial damage [16, 17]. NET-related histones trigger innate immunity by activating TLRs and the NLR-pyrin domain containing (NLRP3) inflammasome. NETs also activate caspase-1, the enzyme of the inflammasome, leading to the release of active IL-1β and IL-18 [18]. Furthermore, NETs combine with complement 1q to activate the classic pathway of complement, consuming a large amount of complement, while activated complements can inhibit the degradation of NETs and aggravate the autoimmunity. A part of lupus glomerulonephritis can be observed to have local NETs formation, and NET-related histone release elicits cytotoxic and immunosimulatory effects [19]. The suboptimal clearance of SLE is associated with disease activity, which is believed to be the result of activation of germinal center B cells [20]. A recent study has found that NETs contain ubiquitinated proteins, one of the translated modified proteins, which can involve in autoimmunity. Specifically, K63 ubiquitination is involved in DNA repair, signaling through NF-κB and endosomal traffic regulation, all of which are related to the modulation of immune responses [21]. In fact, there is a decrease in ubiquitination in NETs from subjects with SLE patients and, in the case of NETosis, it leads to more serious oxidative damage [22].

## Dysregulation of adaptive immunity

### T cells

Breakdown of immune tolerance is critical in the development of SLE and T cells play an important role in this process. In addition to showing abnormal cytokine secretion and cell signal transduction, it can also lead to inappropriate recruitment and activation of B cells and DCs in inflammatory sites [23].

#### T-cell signaling alteration

##### T-cell receptor (TCR)-CD3 signaling pathway

**Fig. 2:**
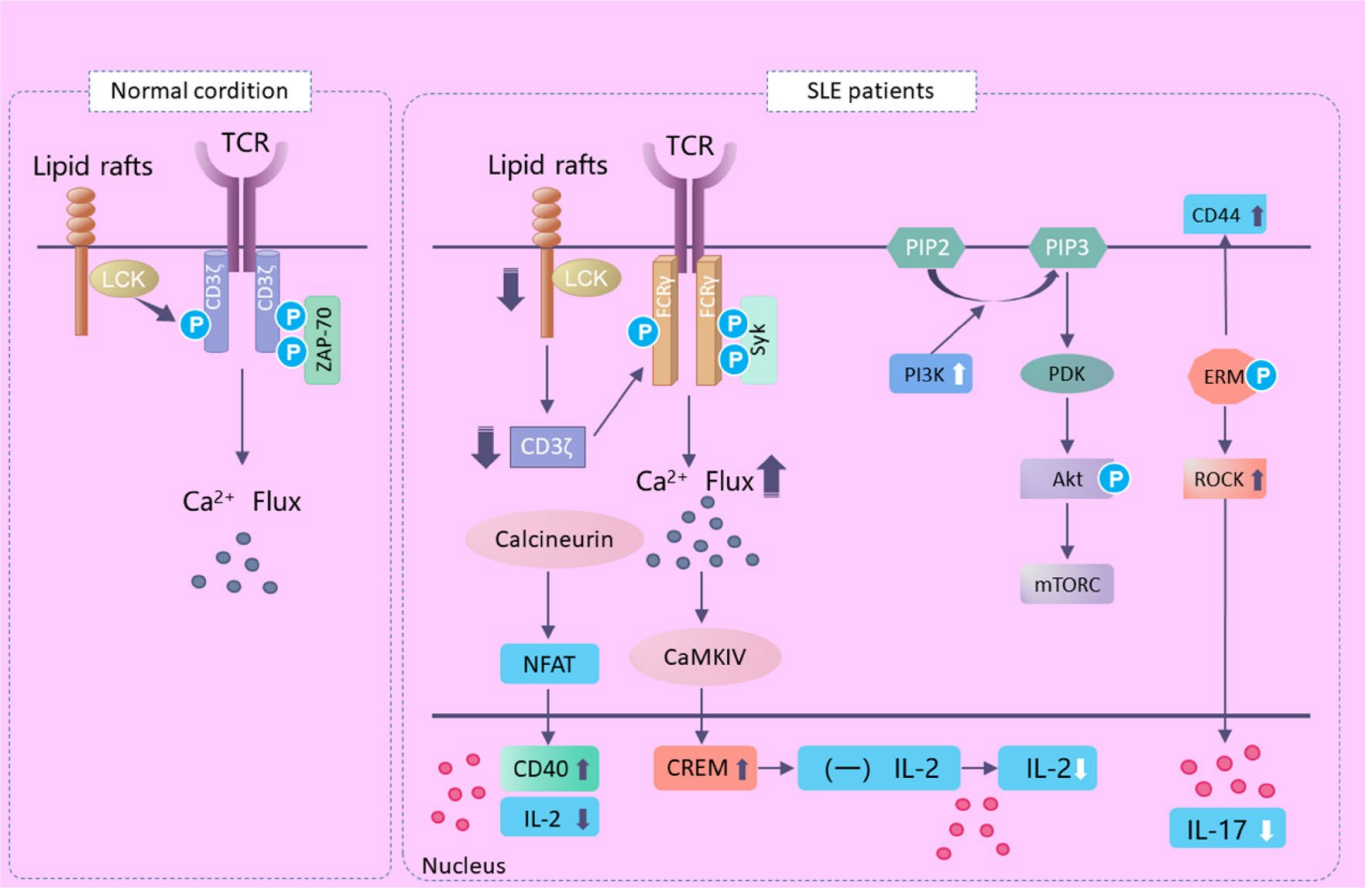
T-cell signaling alteration in systemic lupus erythematosus (SLE). *TCR* T cell receptor, *Lck* lymphocyte-specific protein tyrosine kinase, *ZAP*-*70* zeta-chain-associated protein kinase 70, *FcRγ* Fc receptor common gamma subunit chain, *Syk* Spleen tyrosine kinase, *NFAT* nuclear factor of activated T cells, *CaMKIV* activated calmodulin kinase IV, *CREM* cAMP responsive element modulator, *IL* interleukin, *PIP2* phosphatidylinositol-4, 5-bisphosphate, *PIP3* phosphatidylinositol-3, 4, 5-triphosphate, *PI3K* phosphoinositide-3 kinase, *Akt* protein kinase B, *mTORC* mammalian target of rapamycin complex, *ERM* ezrin/radixin/moesin, *ROCK* Rho-associated protein kinase

CD3 is a marker expressed on the surface of mature T cells, which forms the TCR-CD3 complex in a non-covalent bond with TCR, and participates in the immune response to antigen stimulation. CD3ζ is the main signaling molecule in the TCR-CD3, which contains immunoreceptor tyrosine-based activation motif (ITAM) domains. Lck, the Src kinase lymphocyte-specific protein tyrosine kinase, phosphorylates ITAMs of CD3ζ following TCR recognition and engagement of the MHC-antigen complex. Phosphorylated CD3 ITAMs recruit the ζ-associated protein kinase 70 (ZAP-70); Lck phosphorylates and activates ZAP-70, resulting in calcium influx into T cells [24]. In SLE, the expression of CD3ζ chain was significantly decreased, leading to the recompilation of TCR complex, and CD3ζ was replaced by the homologous Fc receptor common gamma subunit chain (FcRγ) [25]. FcRγ recruits the spleen tyrosine kinase, resulting in the higher calcium influx into T cells. Heightened calcium responses lead to increased activation of calcineurin. Calcineurin dephosphorylates inactive cytoplasmic nuclear factor of activated T cells (NFAT) and dephosphorylated NFAT translocates to the nucleus. Therefore, promoter of CD40L gene as well as T cells become more easily to be activated. On the other hand, activated calmodulin kinase IV increases the expression of intronuclear cAMP responsive element modulator α and inhibits the production of IL-2. Lck localizes to lipid rafts, while accumulation of lipid rafts can aggravate the condition of the lupus mouse in previous studies [26]. Further research shows that T cells isolated from SLE patients have higher levels of ganglioside M1 and cholesterol, a component of the lipid raft domain, which confirmed that lipid rafts play an important role in the pathogenesis of SLE (Fig. 2).

##### CD44–Rock–ERM signaling pathway

CD44 is a cell surface molecule involved in T cell activation and adhesion. Enhanced expression of CD44 was found in SLE patients of which splicing variants CD44V3 and CD44V6 were correlated with disease activity [27–29]. The role of CD44 in adhesion and migration requires interaction with the ezrin/radixin/moesin (ERM) proteins. Rho-associated protein kinase (ROCK) is a serine/threonine kinase that phosphorylates the ERM protein. In patients with SLE, the phosphorylation level of ERM protein increased, and T-cell adhesion and migration were enhanced in patients with SLE. In addition, ROCK can also activate IRF4, affect the differentiation of Th17 and control the production of IL-17 and IL-21 [30].

##### PI3K-Akt-mTOR signaling pathway

Phosphoinositide-3 kinase (PI3K) is a member of the lipid downstreams kinase family. At present, class I PI3K is the most studied one. It can be activated by G protein coupled receptor and receptor tyrosine kinase. Akt is an important target kinase downstreamed PI3K. The mammalian target of rapamycin (mTOR) is a central regulator integrating nutritional information, and mTOR signal activation increases protein synthesis.

Studies in humans and animals have found that the activity of PI3K is enhanced in SLE, while the application of PI3K inhibitors can reduce inflammation in tissues and relieve clinical symptoms [31, 32]. PI3Ks are recruited to the TCR complex following activation and generate phosphatidylinositol-3,4,5-triphosphate (PIP3) from membrane phospholipids. Phosphoinositide-dependent protein kinase 1 then phosphorylates and activates Akt through PIP3. Phosphorylated Akt activates mTOR and promotes the synthesis of proteins, which participate in T-cell division, proliferation and survival (Fig. 2). Previous studies have shown that Akt expression is up-regulated in T and B cells in peripheral blood of SLE, and the level of Akt/mTOR activation in B cells is positively correlated with the severity of the disease [33, 34]. Recently, Borlado et al. [35] found that the active form of PI3K in P65^PI3K^Tg mice formed lupus-like renal changes.

#### T-cell subsets and imbalance

##### Imbalance of Th1/Th2 cells

Th cell is a subtype of CD4+ T cells. Dysfunction of Th cells is closely related to the occurrence and development of SLE. And the imbalance of Th1/Th2 cells is considered to be an important part in the pathogenesis of SLE.

Th cells can be divided into Th1 and Th2 cells according to the secretion of different cytokines (the function of several cytokines, see Table 1) and the adjustment process is a dynamic process. Th1 cells secrete tumor necrosis factor-α (TNF-α), IL-2, IFN-γ, which involve in the activation of macrophages and CD8+ T cells, associated with organ-specific autoimmune diseases. Th2 cells secrete IL-4, IL-6, and IL-10, which can promote the activation of B lymphocytes and induce the production of IgG1. Under normal circumstances, the two types of cells regulate and inhibit each other through cytokines to maintain the immune balance. In SLE, the above balance is broken, but the tendency of the balance is still controversial at present. The most scholars believe that active SLE is characterized by decreased function of Th1 and hyperfunction of Th2, which leads to excessive activation of B cells, generation of autoantibodies and tissue injury [36, 37]. However, Dolff et al. [38] found that Th1 dominated the balance of Th1/Th2 in the chronic course of SLE, especially in patients with lupus nephritis IV. Further studies are needed.

**Table 1:** Several important cytokines in the balance of Th1/Th2 in SLE

Cytokines	Source	Physiological effect	Trends in SLE	Functions in SLE
IL-2	Activated T cells and NK cells	To induce T cell proliferation and maintain immune tolerance	↓	The decrease of IL-2 promotes T cells to differentiate into Th17, inhibits apoptosis and activates autoimmune T cells
IL-4	Th2 cells	Promote humoral immunity, inhibit cellular immunity	↓	Promote the production of IgG dsDNA antibody; promote the occurrence of lupus nephritis
IL-6	Macrophage, T and B lymphocytes and other immune cells	Participate in inflammatory response and immune response	↑	B cells express high levels of IL-6R and bind to IL-6 to promote B cells to produce IgG and dsDNA, resulting in immune damage
IL-10	Macrophage, monocyte, T and B lymphocytes	It has both anti-inflammatory and inflammatory functions	↑	Promote the activation and proliferation of B cells while inhibiting the function of APC and Th1
IFN-γ	Activated T cells and NK cells	Regulate immune response and fight tumors	↑	Promote the activation of B cells
TNF-α	Mononuclear macrophages	Involved in the inflammatory process of autoimmunity	↑	Promote the expression of major histocompatibility complex antigens, producing immune responses

*Th* T helper, *SLE* systemic lupus erythematosis, *IL* interleukin, *NK* natural killer, *IFN* interferon, *TNF* tumor necrosis factor, *IgG* immunoglobulin G, *APC* antigen presenting cell

##### Imbalance of Th17/Treg cells

Th17 cells, a subset of effector CD4+ T cells, are identified based on their ability to produce IL-17A, IL-17F and IL-22, mediating inflammatory responses and participate in the occurrence of autoimmune diseases. IL-17 is the main cytokine that promotes Th17 to participate in SLE. It has been confirmed that the level of IL-17 in the kidney of patients with lupus nephritis is increased, and the gene expression of IL-17 in urinary sediment is increased too [39, 40]. Furthermore, the expression of Th17 was also found in the skin, lung and kidney tissues of SLE patients. And increased Th17 was associated with disease activity in SLE. IL-17 and B-cell stimulating factor (BLys) working together to up-regulate the differentiation and survival of B cells, thereby up-regulating humoral immunity to produce autoantibodies.

Regulatory T (Treg) cells are involved in self-tolerance and their impaired function is associated with the development of autoimmunity. Tregs are capable of modulating the function of effector T cells, maintaining immunological homeostasis, and preventing autoimmunity. Several studies have found that the number of Tregs in SLE patients is reduced while the function is absent [41, 42]. Under normal conditions, Th17 and Tregs are in a state of dynamic equilibrium, which is destroyed in SLE [43]. Currently, it is generally believed that the increase of Th17 cells is accompanied by the decrease of Tregs and the dynamic changes of both are involved in the immune response process.

##### T follicular helper (Tfh) cells

Tfh cells, a T helper-cell subset assisting B cells in germinal centers (GCs), play a major role in GC formation and the selection of high-affinity B cells. Multiple evidences show that Tfh cells and GC responses are associated with the occurrence of SLE. The Tfh cell phenotype in the circulation (cTfh) of patients with SLE is composed as follows: chemokine receptor CXCR3 and CCR6, inducible co-stimulator (ICOS), programmed death molecule-1 (PD-1). ICOS delivers activation signals to CD4+ T cells when these cells interact with APCs, while PD-1 delivers inhibitory signals. Blocking the ICOS ligand in NZB/NZW mice can inhibit the function of Tfh and the formation of GC, and reduce the anti-dsDNA antibody titers [44]. After co-culture of naive B cells with cTfh in vitro, cTfh can produce IL-21, induce B-cell proliferation and differentiation, and produce IgG and IgA. In vivo experiments showed that increased cTfh cells were positively correlated with the disease activity and serum autoantibody titers [45, 46]. Recent study suggests that immune complexes containing nucleic acid also promote the generation of autoantibodies by enhancing Tfh-cell responses in SLE, which further proved that Tfh is involved in the pathogenesis of SLE.

### B cells

There is abnormal central and peripheral tolerance of B cells in SLE patients. A large number of self-reactive B cells produce variety of autoantibodies leading to the occurrence of lupus.

#### Classic T–B cells interactions

**Fig. 3:**
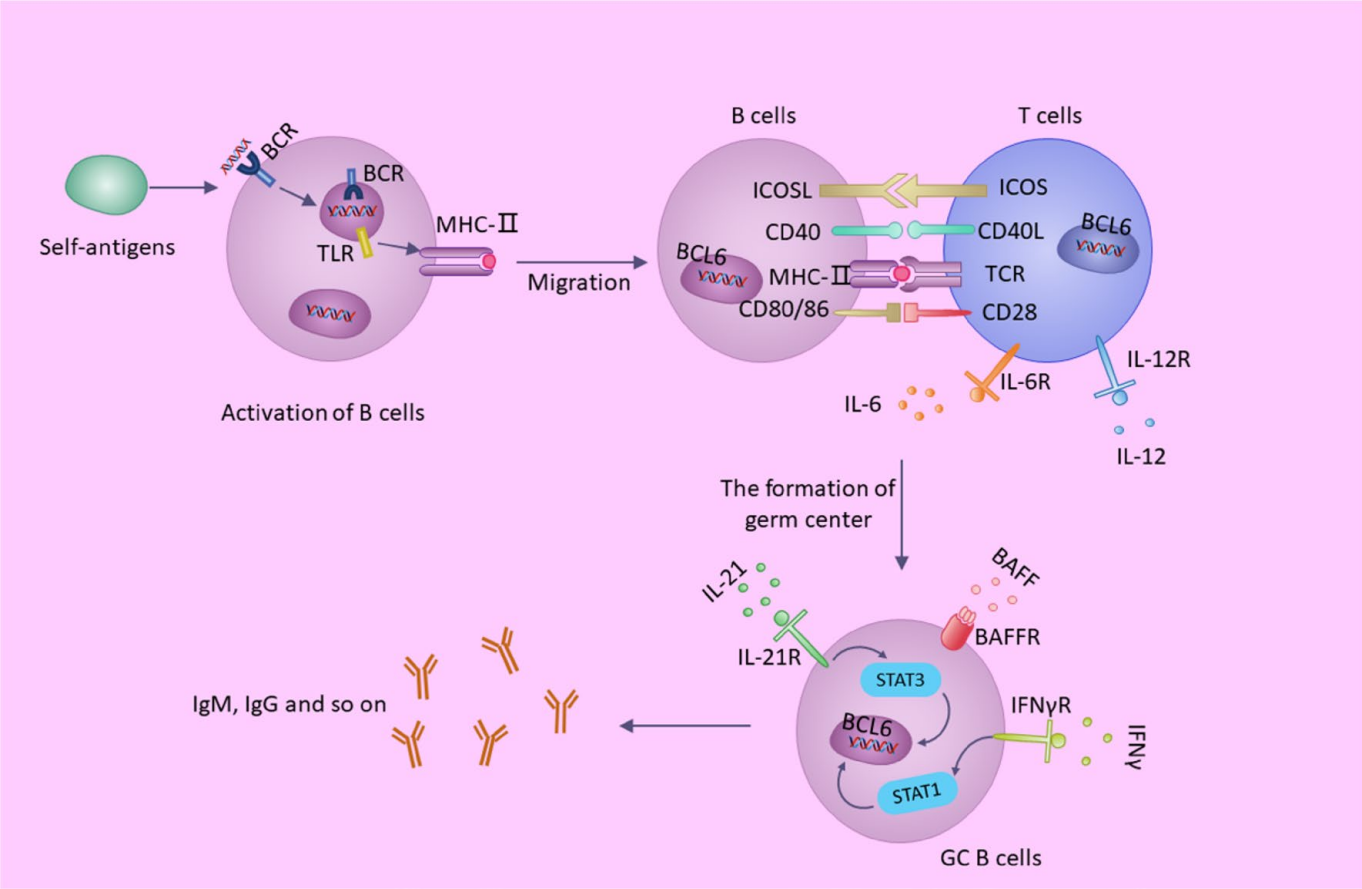
Classic T–B cells interactions in SLE. *BCR* B-cell receptor, *TLR* Toll-like receptor, *MHC*-*II* major histocompatibility complex-II, *BCL* B-cell lymphoma, *ICOS* inducible co-stimulator, *ICOSL* inducible co-stimulator ligand, *TCR* T cell receptor, *IL* interleukin, *IgM* immunoglobulin M, *IgG* immunoglobulin G, *BAFF* B-cell activating factor, *BAFFR* B-cell activating factor receptor, *IFNγ* interferon γ, *IFNγR* interferon γ receptor, *GC* germinal center

Immature B cells show a unique activation tendency through the integration signals downstream of B-cell receptor (BCR) and TLRs. BCR recognizes specific antigens containing RNA and/or DNA and forms protein polypeptides after TLR management, then activating B cells. The activated B cells migrate to the boundary of the follicle and interact with CD4+ T cells through TCR and co-stimulators. On the one hand, activated B cells secrete cytokine IL-6, TNF, IFN-γ and IL-10 [47]. On the other hand, activated CD4+ T cells migrate to B-cell follicles to produce IL-21 and IFN-γ as Tfh cells. Tfh cells promote GC formation through the production of IL-21, which sustains the expression of B-cell lymphoma 6 (BCL-6) and promotes B-cell activation, class-switch recombination and plasma cell differentiation. Long-lived plasma cells are eventually formed (Fig. 3).

B-cell activating factor (BAFF) is also involved in T–B cells interactions. BAFF is overexpressed, which promotes the proliferation of B cells and prolongs the survival time of self-reactive B cells. BAFF transgenic mice showed severe B-cell proliferation, anti-ds DNA antibody formation, serum IgM, IgA, IgE and IgG elevation, and renal tissue showed lupus-like changes such as immune complex deposition [48]. Moreover, studies in NZB/NZW lupus mice showed an increase in BAFF in the early stage of SLE, and BAFF serum content was positively proportional to the degree of renal injury.

#### B-cell subsets

Regulatory B cells (Bregs) are a group of cells with negative regulation of the immune response. Its immunomodulatory effect mainly depends on the secretion of IL-10 and transforming growth factor β. IL-10 can inhibit the production of pathogenic Th1 cytokines, affect the activation and apoptosis of B lymphocytes, and regulate antigen presentation, playing an important role in autoimmune regulation and inflammation. Studies in mice showed that Bregs produced IL-10, which regulates the T-cell-dependent immune response [49]. A growing number of human SLE studies showed that Bregs in patients with lupus were lower than in healthy people, especially in patients with lupus nephritis, and the number of these cells increases after immunosuppressive treatment [50]. In addition, the response of Bregs to CD40 stimulation and the secretion of IL-10 were reduced in peripheral blood of SLE patients, which indicate that Bregs in SLE had dysfunction [51].

## Potential therapeutic targets in SLE

The treatment guidelines for SLE were established from American College of Rheumatology 1999 to European League Against Rheumatism 2008, and no new drugs were added in these years. The cornerstones of SLE therapies now are non-steroidal anti-inflammatory drugs, glucocorticoids, hydroxychloroquine, and immunosuppressive agents. However, all of these treatments are aimed at controlling the symptoms of the disease and do not address the underlying cause; they have a broad range of nonspecific effects and are associated with considerable toxicities. With the deepening understanding of the pathogenesis of SLE, drugs targeted at possible links of the disease have been studied, providing a new direction for the treatment of SLE.

### Targeting innate immunity

The advances in the understanding of the molecular basis of innate immunity have led to the identification of IFNs, particularly IFN-α, as key mediators in the pathogenesis of SLE. Targeting of IFNs (Table 2), therefore, has emerged as important developments for novel drug research in lupus.

**Table 2:** Agents targeting innate immunity

Type	Agents	Targets for agents	Clinical evaluation
IFN targeting therapies	Rontalizumab	Anti-IFNα	Did not meet the end point
	Sifalimumab	Anti-IFNα	No clinical benefits with AE of herps zoster
	AGS-009	Anti-IFNα	Safe and well tolerated in phase I RCT
	JNJ-55920839	Anti-IFNβ	Phase I RCT is ongoing
	Anifrolumab	Anti-IFNAR	Safe and effective in reducing IFN signature
	AMG-811	Anti-IFNγ	Safe and well tolerated with no significant benefits
	IFN-K	IFNα inhibition	Safe and well tolerated with no significant benefits
pDC targeting therapies	BIIB059	Anti-BDCA2	Safe and effective in reducing IFN signature
	JNJ-56022473	Anti-CD123	Phase I RCT is ongoing
	ABT-199	Bcl-2 inhibitor	Outcome of phase I trial is pending

*RCT* randomized controlled trial, *IFN* interferon, *pDC* plasmacytoid dendritic cell, *BDCA* blood dendritic cell antigen, *Bcl* B-cell lymphoma, *AE* adverse events

Anifrolumab is a fully human IgG1κ monoclonal antibody directly against the subunit 1 of the type I interferon receptor. It has been assessed in phase I and phase II clinical trials. The phase II study [52] showed that the effect size was larger in patients with a high IFN signature at baseline in patients treated with anifrolumab. There was no significant difference in adverse events except for Herpes zoster. Further studies are ongoing in active SLE patients, including in lupus nephritis (LN) (NCT02446899, NCT02547922).

Rontalizumab and sifalimumab, unlike anifrolumab, are anti-IFNα drugs that have been tested in SLE patients. SLE patients treated with rontalizumab showed some efficacy in patients with low IFN signature; however, it did not meet the primary end points [53]. Sifalimumab also showed no efficacy. Neither of them entered further development stages. AGS-009, JNJ-55920839 and AMG-811 are new anti-IFN monoclonal antibodies being assessed in phase I clinical trials.

IFN-a-kinoid (IFN-K), a vaccine composed of IFNa2b coupled to a carrier protein, is another option in blocking IFN-α. It acts by inducing antibody production against all IFNα subtypes. Current study showed that IFN-K significantly reduced the expression of the IFN signature compared to placebo, but with no differences in disease activity scores, serum C3, C4 or anti-ds-DNA concentrations.

Plasmacytoid dendritic cells (pDCs) have the most potent capacity to produce IFNα of any IFN-producing cell. The treatment option targeting pDCs directly have, therefore, long been expected in SLE. Alternative strategies targeting the pDCs (Table 2) include the use of anti-blood dendritic cell antigen 2 antibody, anti-CD123 monoclonal antibodies and BCL-2 inhibitors, which were assessed in different phase of clinical trials.

### Targeting adaptive immunity

#### T-cell target therapies

B cells require T cell help to produce high-affinity IgG autoantibodies. Distinct signals are necessary for the activation of T cells. The second signal can be activated via co-stimulatory signals, including CD40/40L, CD28, cytotoxic T-lymphocyte antigen 4 (CTLA-4), CD80/CD86 and ICOSL/ICOS.

Abatacept is a fusion protein composed of the Fc region of the immunoglobulin IgG1 fused to the extracellular domain of CTLA-4, which has a higher affinity with CD80/CD86 than CD28. It failures the activation of T cells by blocking the co-stimulation of T and B lymphocytes, and further preventing B-cell response [54]. The effect of this drug in arthritis is clear, but the effect in SLE remains unclear. In a phase II/III clinical trial [55], abatacept demonstrated efficacy in increasing C3 and C4, accompanied by reducing ds-DNA levels. However, subsequent RCT focusing on LN [56] did not meet the primary end points.

CD40-CD40L is another important receptor/ligand pair required for Tfh cell differentiation. BG9588 is anti-CD40L monoclonal antibodies (mAb). Early trial with BG9588 was discontinued because of thromboembolic events. New anti-CD40L mAb, dapirolizumab, is being assessed (NCT02804763). More agents against receptor/ligand pair see Table 3.

**Table 3:** Agents targeting adaptive immunity

Type	Mechanism of action	Agents	Targets for agents	Clinical evaluation
B-cell targeting therapies	Blocking B-cell surface antigen	Rituximab	Anti-CD20	Effective in refractory SLE. Allergic response is the main AE
		Ofatumumab	Anti-CD20	Effective in increasing C3 and C4, decreasing anti-dsDNA. Infection is the main AE
		TRU-015	Anti-CD20	Phase I RCT is terminated
		Obinutuzumab	Anti-CD20	Safe and well tolerated in phase I trial
		Epratuzumab	Anti-CD22	Phase III trial did not meet the primary end points
		SM03	Anti-CD22	Safe and well tolerated
		XmAb 5871	Anti-CD19	Phase II trial is ongoing
		Milatuzumab	Anti-CD74	Outcomes of phase I trial is pending
	Blocking B-cell survival factors	Belimumab	Anti-BAFF	Effective in improving time to first flare and exhibiting a steroid sparing effect
		Blisibimod	Anti-BAFF	Effective in increasing C3 and C4 levels and decreasing urinary protein
		Atacicept	Anti-BAFF/APRIL	Got Several efficacy, but trials were terminated for fatal infections
	B-cell tolerization	Abetimus sodium	Anti-immunoglobulin receptor on B cells	Clinical trials were terminated
T-cell targeting therapies	Blocking co-stimulatory factors	Abatacept	Competing with CD28	Effective in increasing C3 and C4, decreasing anti-dsDNA levels
		BG9588	Anti-CD40	Clinical trials were terminated for thromboembolic event
		Dapirolizumab	Anti-CD40L	Safe and well tolerated in phase I trial
		MEDI-570	Anti-ICOS	Outcomes of phase I trial is pending
		AMG557	Anti-ICOSL	Outcomes of phase I trial is pending
		Theralizumab	Anti-CD28	Phase II trial is ongoing

*BAFF* B-cell activating factor, *APRIL* a proliferation-inducing ligand, *ICOS* inducible co-stimulator, *ICOSL* inducible co-stimulator ligand, *SLE* systemic lupus erythematosis, *AE* adverse events, *RCT* randomized controlled trial

#### B-cell target therapies

The hallmark of immunological abnormalities in SLE is loss of B-cell tolerance, increasing the production of a variety of autoantibodies that against nuclear antigen. Therefore, novel therapeutic agents are developed to target at the growth/survival factors, surface molecules and receptors of B cells, leading to their apoptosis, depletion or anergy.

Belimumab is a human IgG1λ mAb that inhibits B-cell survival and differentiation by blocking the soluble BLys, which is also called BAFF. It was approved in the USA and Europe in 2011 for the treatment of adults with active autoantibody-positive SLE already receiving standard therapy. According to the phase III RCTs, the drug demonstrated a greater therapeutic response in a subpopulation of patients characterized by having higher baseline disease activity as defined by anti-dsDNA positivity, hypocomplementemia (C3 or C4), or corticosteroid treatment requirement [57]. A proliferation-inducing ligand (APRIL) shares similar functions with BAFF and is also important for the survival and activation of B cells [58]. Unlike BAFF, which binds to BAFF receptor only, APRIL binds to transmembrane activator-1 and calcium modulator ligand interactor (TACI), and B-cell maturation antigen receptors with a higher affinity than BAFF. Studies in vivo showed that TACI-Ig (but not BAFF-R-Ig) can suppress serum IgM antibodies, reduce plasma cell frequency in the spleen and inhibit IgM responses to a T-cell-dependent antigen [59]. Atacicept is the representative of TACI-Ig agent. Other B-targeted agents are also under evaluation (Table 3).

Rituximab is a human–mouse chimeric mAb against CD20 receptors that can induce the apoptosis of B cells, inhibit the proliferation of B cells, and effectively remove the abnormal proliferation of B cells. It was first marketed and approved by the FDA to treat B-cell lymphomas and showed significant clinical benefits. Subsequent studies demonstrated that rituximab is effective in refractory SLE manifestations, including nephritis and neuropsychiatric disease in both adult and pediatric patients [60–63]. The main adverse event is allergic response because it is a chimeric anti-CD20 antibody. Other anti-CD20 antibodies, including the fully human mAb (ofatumumab) and engineered versions with improved antibody-dependent cellular cytotoxicity killing (obinutuzumab), are investigated in the SLE pipeline [64]. Because of the similar rationale to target B-cell-specific surface antigens that are expressed broadly across different B-cell subsets, therapeutics against CD22, CD19 and CD74 are also being explored (details see Table 3).

### Other target therapies

The immunological pathogenesis of SLE is complex. The innate and acquired immune networks are interlinked with each other mutually complementary, so any point cannot exist independently. Cytokines, complements, immune complexes and kinases of the intracellular machinery play important roles in SLE. Agents targeting them have potential therapeutic effects and many clinical trials are ongoing (details see Table 4). Several other strategies, not discussed here, target on proteasome inhibitor, spleen tyrosin kinase inhibitor, Janus kinase inhibtor, sphingosine 1-phosphate receptor modulator, Calgranuline B modulator and chaperonin 10 might also prove of value.

**Table 4:** Other specific targeting agents

Type	Agents	Targets for agents	Clinical evaluation
Targeting cytokines	Tocilizumab	Anti-IL-6 receptor	Effective in increasing C3 and C4, decreasing anti-dsDNA levels
	Etanercept	Anti-TNF	Safe and well tolerated
	BT063	Anti-IL-10	Phase II trial is ongoing
Targeting complement	Eculizumab	Anti-C5	Randomized trials are ongoing
Targeting IC	SM101	Anti-IC	Safe and well tolerated, no serious AE
Targeting kinases of the intracellular machinery	Evobrutinib	Anti-BKT	Randomized trials are ongoing

*IC* immune complexes, *IL* interleukin, *TNF* tumor necrosis factor, *AE* adverse events, *BKT* Bruton’s tyrosine kinase

## Conclusions

**Fig. 4:**
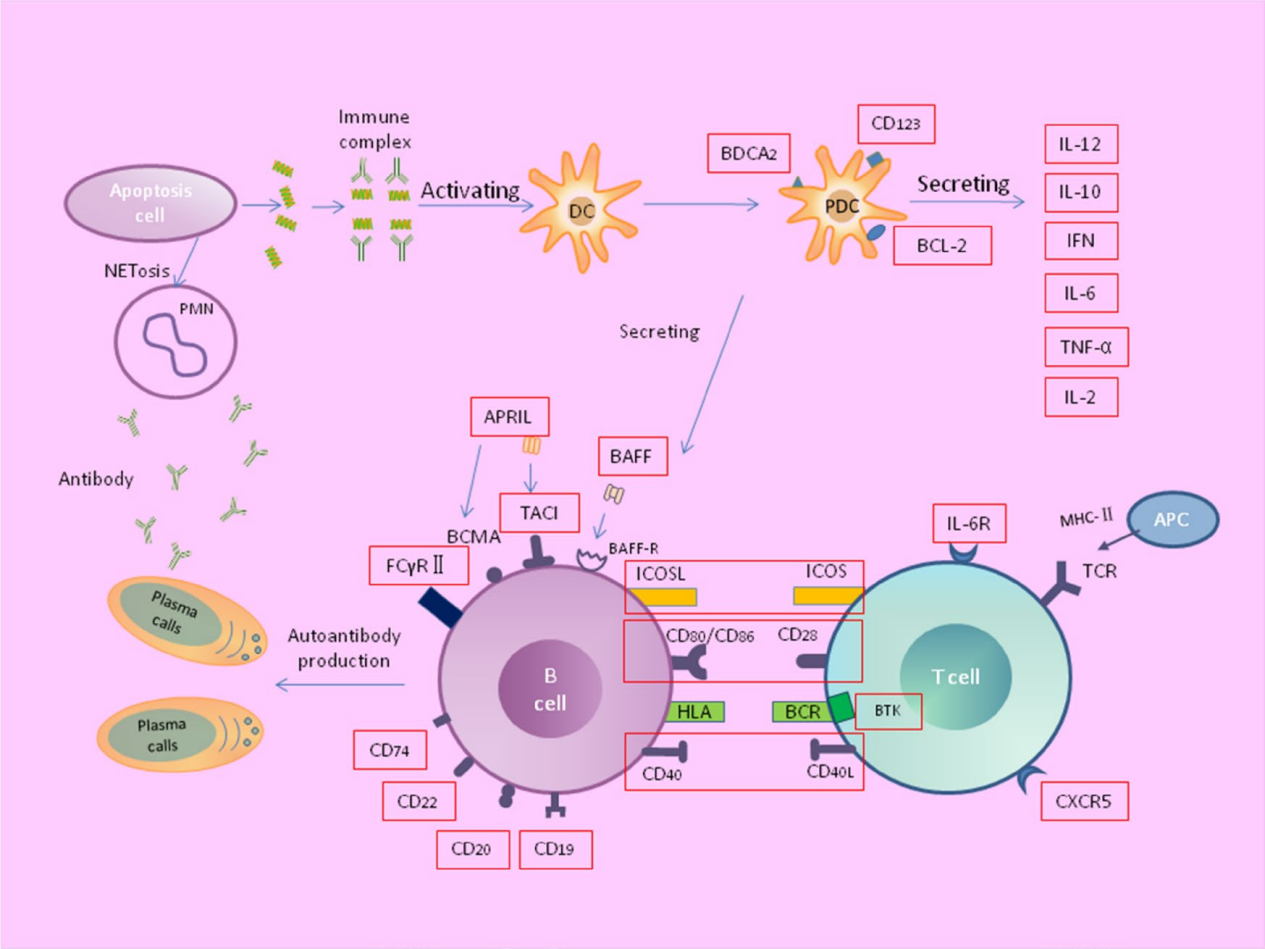
The immune pathogenesis of SLE and targets of SLE treatment (treatment targets are marked by red boxes). *NETosis* neutrophil extracellular trap formation, *PMN* polymorphonuclear neutrophils, *DC* dendritic cell, *BDCA* blood dendritic cell antigen, *BCL* B-cell lymphoma, *IL* interleukin, *IFN* interferon, *TNF* tumor necrosis factor, *APRIL* a proliferation-inducing ligand, *BAFF* B-cell activating factor, *BCMA* B-cell maturation antigen, *TAIC* transmembrane activator-1 and calcium modulator ligand interactor, *ICOS* inducible co-stimulator, *ICOSL* inducible co-stimulator ligand, *HLA* human leukocyte antigen, *BCR* B-cell receptor, *BTK* Bruton’s tyrosine kinase, *MHC* major histocompatibility complex, *TCR* T cell receptor, *CXCR5* CXC chemokine receptor type 5

SLE is a multifactorial and complex autoimmune disease, which is characterized by various cellular and molecular aberrations. The pathogenesis of SLE is still far away to be fully understood. Dysregulated immune response in SLE has been extensively studied, including innate immunity and adaptive immunity. And a dramatic expansion has been achieved in our understanding of cellular and molecular phenotypes in the pathogenesis of SLE. B lymphocyte plays a central role in adaptive immune response of SLE, which involved in the production of autoantibodies, presentation of autoantigens and activation of autoreactive T cells. Furthermore, T lymphocyte plays a role through co-stimulator-mediated signaling pathway and cytokines secreted by subsets of T cells. The role of innate immune response in SLE pathogenesis has also been noticed, especially the discovery of TLR on pDC that can be activated by immune complex, inducing the production of IFN-α and the formation of NETs. These abnormalities co-exist and complement each other. Figure 4 shows the link between these factors and the sites of action of relevant therapeutic targets. Understanding the immune pathophysiology of SLE has led to the emergence of new biologic agents that aim to specifically target abnormal immune processes and thus reduce the unwanted adverse events associated with conventional broad-spectrum immunosuppressant therapies. Even though our understanding of SLE remains incomplete and most of the novel drugs are in clinical trials, these new biologic agents and small-molecule drugs may culminate in the development of safer and more effective therapies.
